# Greater Independence in Activities of Daily Living is Associated with Higher Health-Related Quality of Life Scores in Nursing Home Residents with Dementia

**DOI:** 10.3390/healthcare3030503

**Published:** 2015-03-30

**Authors:** Charice S. Chan, Susan E. Slaughter, C. Allyson Jones, Adrian S. Wagg

**Affiliations:** 1 Faculty of Agricultural, Life and Environmental Sciences, University of Alberta, Edmonton, AB T6G 2R3, Canada; E-Mail: charice@ualberta.ca; 2 Faculty of Nursing, University of Alberta, Edmonton, AB T6G 2R3, Canada; 3 Department of Physical Therapy, Faculty of Rehabilitation Medicine, University of Alberta, Edmonton, AB T6G 2R3, Canada; E-Mail: cajones@ualberta.ca; 4 Department of Medicine, Faculty of Medicine and Dentistry, University of Alberta, Edmonton, AB T6G 2R3, Canada; E-Mail: wagg@ualberta.ca

**Keywords:** activities of daily living, health-related quality of life, dementia, Functional Independence Measure, Resident Assessment Instrument-Minimum Data Set

## Abstract

Health-related quality of life (HRQL) for nursing home residents is important, however, the concept of quality of life is broad, encompasses many domains and is difficult to assess in people with dementia. Basic activities of daily living (ADL) are measured routinely in nursing homes using the Resident Assessment Instrument-Minimum Data Set Version 2.0 (RAI-MDS) and Functional Independence Measure (FIM) instrument. We examined the relationship between HRQL and ADL to assess the future possibility of ADL dependency level serving as a surrogate measure of HRQL in residents with dementia. To assess ADL, measures derived from the RAI-MDS and FIM data were gathered for 111 residents at the beginning of our study and at 6-month follow-up. Higher scores for independence in ADL were correlated with higher scores for a disease-specific HRQL measure, the Quality of Life—Alzheimer’s Disease Scale. Preliminary evidence suggests that FIM-assessed ADL is associated with HRQL for these residents. The associations of the dressing and toileting items with HRQL were particularly strong. This finding suggests the importance of ADL function in HRQL. The RAI-MDS ADL scales should be used with caution to evaluate HRQL.

## 1. Introduction

The maintenance or improvement of health-related quality of life (HRQL) is important in the treatment of persons with dementia [1]. Applications of HRQL assessments in a nursing home environment can include monitoring for quality improvement, surveying of perceived population health, medical auditing, conducting clinical trials and cost-utility analyses [2]. The multidimensional nature of HRQL creates a challenging task of defining and assessing it in this population [3]. HRQL can include symptoms, mental health, physical functioning, role functioning and overall perception of health. A central feature of HRQL measures is the inclusion of a patient or proxy respondent’s evaluation of health that would not otherwise be captured. Several factors make it challenging to measure HRQL in people with dementia including language barriers, anosognosia, neuropsychiatric symptoms, and impaired cognition [4].

With varying operational definitions of HRQL, a wide variety of measures exist that assesses fundamentally different constructs. When many HRQL instruments are used there may be confusion as to what is really being assessed. There have been attempts to determine which domains most accurately measure HRQL [5,6] but further research is still needed to agree upon a standard definition.

The taxonomy of HRQL includes generic or disease-specific measures yet methodological concerns still exist. For example, Ettema *et al.* [7] examined generic measures, such as the World Health Organization Quality of Life 100 (self-report measuring aspects such as psychological function, physical state, religion, and environment), Nottingham Health Profile (mostly caregiver reports measuring aspects such as mobility, pain and sleep), and Health Utility Index 2 (caregiver reports measuring aspects such as sensation, mobility, and cognition), and found that reliability of the instruments was not always reported and when it was, it was insufficient. Furthermore, these authors [7] only found reports of responsiveness for two dementia-specific HRQL measures (Dementia Care Mapping and Alzheimer’s Disease Related Quality of Life). A determination of which instrument might be most applicable to certain situations has been attempted [8], but a study by Sloane *et al.* [9] claimed that a combination of measures should provide the best assessment of HRQL. The existence of many generic and disease specific quality of life instruments worldwide, with varying domains, lengths, and reliabilities, demonstrates the difficulty of agreeing on how to define and measure HRQL in a clinical setting [1].

Basic activities of daily living (ADL), such as dressing, eating, toileting, and transfers, are outcomes that are more observable and perhaps more relevant to persons with dementia [10,11]. Nursing home leaders may find it convenient to use ADL data from the Functional Independence Measure (FIM) to guide HRQL outcome initiatives. The FIM is collected in nursing homes and rehabilitation centres in many countries, including Canada, USA, Australia, France, Sweden, and Germany [12]. Previous studies have examined the relationship between ADL and HRQL and found correlations between ADL and HRQL. In patients with strokes, ADL was highly correlated with quality of life (QOL) as measured by the FIM [13]. A small positive correlation was found between levels of physical and functional dependence and perceived QOL in veterans with dementia [14]. Studies of frail older adults found that improving ADL disabilities improved participant HRQL [15] and found that the number of ADL limitations was associated with their HRQL [16].

To our knowledge, no studies have examined this relationship using the FIM and Resident Assessment Instrument-Minimum Data Set Version 2.0 (RAI-MDS, interRAI, Baltimore, MA, USA) in a frail older population with dementia. The objective of this study was to perform an exploratory analysis of the relationship between dependency in ADL and HRQL scores in nursing home residents with a dementia diagnosis.

## 2. Methods

### 2.1. Study Sample

The present study is a secondary analysis of the original quasi-experimental Mobility of Vulnerable Elders (MOVE) study [17] which recruited residents with a dementia diagnosis aged 65 and over from 7 nursing homes in Edmonton, Alberta, Canada.

### 2.2. Procedure

Upon obtaining consent and assent, a disease-specific HRQL measure, the Quality of Life-Alzheimer’s Disease (QOL-AD) and disability measure of the assistance required to perform activities of daily living, FIM were administered by trained research assistants via proxy-interviews with health care aides for each resident at baseline (upon recruitment to the study) and at 6-month follow-up. These health care aides provided direct care to the resident and were familiar with the resident’s activities on a daily basis. In contrast, items on the RAI-MDS directly related to ADL were recorded quarterly by health care aides on 7-day tracking forms. These data were then integrated into the final RAI-MDS assessments completed by Registered Nurses. We used the quarterly RAI-MDS assessments administered closest to each resident’s baseline and six-month time points. The FIM was used to validate findings with the RAI-MDS. Data were collected from July 2011 to February 2013. The study was approved by the University of Alberta Health Research Ethics Board.

### 2.3. Measures

The original version of the Quality of Life—Alzheimer’s Disease (QOL-AD) [18] is a 13-item interview for community-dwelling individuals. It has been adapted to a 15-item scale (caregiver version) for persons with dementia in institutional care and has been adapted in several languages [19]. Domains are rated on a Likert scale of 1 (poor) to 4 (excellent). The total score ranges from 15 to 60, with higher scores representing higher psychosocial HRQL. The QOL-AD measures domains, such as the resident’s mood, energy, memory, life as a whole, friends, and ability to do things for fun. Several studies have demonstrated good internal consistency and test-retest reliability [1,20,21].

The RAI-MDS, a comprehensive assessment tool for nursing homes with over 300 items, has demonstrated adequate reliability and validity in institutionalized adults with dementia [22,23,24]. Within the RAI-MDS, three ADL scales have been developed as a summation of individual item scores: The ADL Short, ADL Long, and ADL Hierarchy. Each scale differs in length and complexity and places resident self-performance on a continuum of self-involvement in ADL. Residents are evaluated on performance and not capacity to perform [25]. Three other scales derived from the RAI-MDS data were used to characterize the residents: The CPS, DRS, and CHESS. The Cognitive Performance Scale (CPS) is a hierarchical scale that rates residents on five MDS items such as decision-making and short-term memory with total scores ranging from 0 to 6 [26]. The Depression Rating Scale (DRS) is derived from mood and behavioral domains in the RAI-MDS and rates residents as 0, 1, 2, for 7 questions with total scores ranging from 0 to 14 and higher scores indicating the prevalence of depression symptoms [27]. Finally, the Changes in Health End-Stage Disease, Signs and Symptoms Scale (CHESS) assesses medical instability with total scores ranging from 0 to 5, with 5 indicating very high health instability [28].

The Functional Independence Measure (FIM) is a performance-based measure that evaluates the assistance required to perform ADL. It consists of 18-items each measured with a scale of 1 (Dependent) to 7 (Independent). Total FIM scores range from 18 to 126, with higher scores representing greater independence [29]. The FIM has good construct validity and high test-retest reliability [30,31]. In this study individual RAI-MDS and FIM ADL items, that comprised the physical domain of the instruments, were chosen based on a FIM-MDS crosswalk created by Williams *et al.* [32].

### 2.4. Analysis

Descriptive analyses were performed on resident characteristics. Cases with missing QOL-AD scores were removed from analysis. Incomplete or missing cases were compared with the analyzed sample to identify any differences in age, sex depression, cognition or medical instability. Due to small numbers of participants in the independent category, residents’ levels of ADL dependency were recoded into three groups, developed by Granger *et al.* [29]: Independent, Modified Dependence or Complete Dependence. Others have also used this category system [33,34]. This enabled an assessment of differences in HRQL across levels of dependency. The RAI-MDS recoding employed the FIM scale score crosswalk of Velozo *et al.* [35] (Table 1).

**Table 1:** Recoding method for RAI-MDS and FIM level of dependence.

RAI-MDS ADL Self Performance Scale	Recoded RAI-MDS Level of Dependence	FIM Scale	Recoded FIM Level of Dependence
0 (Independent)	Independent	7 (Complete Independence)	Independent
		6 (Modified Independence)	
1 (Supervision)	Modified Dependence	5 (Supervision or Setup)	Modified Dependence
2 (Limited Assistance)		4 (Minimal Contact Assistance)	
		3 (Moderate Assistance)	
3 (Extensive Assistance)	Complete Dependence	2 (Maximal Assistance)	Complete Dependence
4 (Total Dependence)		1 (Total Assistance)	

We analyzed the data using Analysis of Variance (ANOVA) to identify the individual ADL factors associated with lower scores of HRQL at baseline and 6-months later. Change in ADL or change in HRQL over the six-months was not examined as this was not the focus of the paper. To examine the differences in the means of the QOL-AD scores across the three levels of dependency in RAI-MDS items and FIM items, several one-way analyses of variance were completed. Bonferroni *post hoc* tests were performed to correct for the multiple comparisons. Data for residents who scored an 8 (activity did not occur) for the Walk in Room RAI-MDS item were removed from the analysis, as an 8 was not included in the crosswalk.

A multivariate linear regression was also performed to determine the magnitude and direction of level of ADL (FIM and RAI) that explained QOL-AD. ADL Short, ADL Long, ADL Hierarchy, and FIM Total were the independent variables. From the original MOVE study, resident age, sex, cognition (CPS score), depression (DRS score) and medical instability (CHESS score) data were available as covariates. All variables with a *p*-value < 0.20, in a bivariate regression of covariates and the dependent variable QOL-AD, were selected to assess for confounding in further analyses [36]. To assess for confounding, a multivariate regression of the independent variables (ADL scales) and dependent variable (QOL-AD) were performed with a single covariate added each time. Covariates that changed the magnitude of the association between the RAI/FIM ADL scale and QOL-AD by 15% or more were confounders [37,38]. Those confounding variables were then adjusted for in the final multiple regression model of the RAI/FIM ADL scales and QOL-AD.

Pearson’s correlations for each item on both instruments were calculated. Statistical significance was defined at the *p* < 0.05 level.

## 3. Results

### 3.1. Resident Characteristics

Two-hundred and seventy-four residents were recruited for the original study and 111 residents who completed baseline and six-month assessments were used in this analysis. The majority of residents were female (70.8%), and the mean age was 86.3 (SD = 7.27) years. Most residents had moderate cognitive impairment (CPS = 3.12; SD = 1.21), mild depression (DRS = 2.48; SD = 2.45) and low medical instability (CHESS = 0.75; SD = 0.93). Dementia diagnoses abstracted from the health record included 28 (25.2%) residents with Alzheimer’s disease, 21 (18.9%) with mixed dementia, 12 (10.8%) with vascular dementia and 50 (45.1%) with unspecified dementia. Residents with missing QOL-AD scores showed fewer symptoms of depression (*p* = 0.001), however they did not differ in age, sex, cognition, and medical instability (*p* > 0.05).

### 3.2. Analysis of Variance

In general, residents who were able to perform ADL with greater independence had higher mean QOL-AD scores than those with Modified Dependence in ADL (Table 2 and Table 3). Similarly, those with Modified Dependence (requiring supervision or limited assistance) had a higher QOL-AD score than those with Complete Dependence (requiring extensive or total assistance). Significant differences were seen between those who were able to perform the activities independently as compared to those who required assistance. There were fewer items with statistically significant differences between those who required assistance and those who were fully dependent for assistance. More items were statistically significant at six months, which was particularly evident for the FIM measure. For example, the statistically significant differences in QOL-AD means, between the Independent and Complete Dependence groups, at six months for the FIM were in relation to Eating (*p* < 0.001), Grooming (*p* < 0.001), Upper Body Dressing (*p* < 0.001), Lower Body Dressing (*p* < 0.001), Toileting (*p* < 0.001), Bladder Management (*p* < 0.001), Bowel Management (*p* = 0.002), and Toilet Transfers (*p* = 0.01).

**Table 2:** QOL-AD mean scores across three levels of dependency for each RAI-MDS ADL item.

RAI-MDS ADL Item	Baseline QOL-AD Score				6-Months QOL-AD Score			
	Residents independent in ADL Mean (SD) *n*	Residents with modified dependence in ADL Mean (SD) *n*	Residents with complete dependence in ADL Mean (SD) *n*	ANOVA *p*-value df	Residents independent in ADL Mean (SD) *n*	Residents with modified dependence in ADL Mean (SD) *n*	Residents with complete dependence in ADL Mean (SD) *n*	ANOVA *p*-value df
Transfer	41.55 (6.99) 20	37.21 (7.89) 57	38.81 (8.26) 26	0.10 2, 100	39.47 (8.89) 19	37.17 (7.55) 47	35.77 (7.83) 35	0.26 2, 98
Walk in Room	38.71 (7.63) 38	38.16 (8.41) 44	35.67 (7.47) 6	0.69 2, 85	37.34 (8.30) 35	36.17 (7.74) 35	35.64 (5.20) 11	0.75 2, 75
Dressing	43.20 (7.980) 5	39.77 (8.20) 31	37.49 (7.69) 67	0.16 2, 100	45.33 (10.21) 6	39.68 (7.10) 19	35.83 (7.49) 76	0.01 ^†^ 2, 98
Eating	40.96 (7.16) 23	38.29 (8.07) 69	34.27 (7.16) 11	0.07 2, 100	40.23 (7.53) 26	36.95 (8.21) 56	33.37 (5.98) 19	0.02 ^†^ 2, 98
Toilet Use	41.47 (5.76) 15	38.84 8.95) 25	37.59 (7.86) 63	0.23 2, 100	45.43 (9.68) 7	39.20 (6.20) 20	35.77 (7.67) 74	0.003 ^†^ 2, 98
Personal Hygiene	47.50 (2.12) 2	39.69 (8.15) 32	37.62 (7.74) 69	0.13 2, 100	47.00 (12.08) 4	41.59 (6.39) 17	35.68 (7.38) 80	0.001 ^†,‡^ 2, 98
Bathing ^a^	0 residents	5 residents	98 residents	N/A	0 Residents	10 residents	89 residents	N/A
Bowel Continence	39.50 (7.16) 28	38.81 (8.09) 27	37.65 (8.32) 48	0.60 2, 100	40.43 (8.16) 30	34.82 (7.53) 22	36.12 (7.47) 49	0.02 ^§^ 2, 98
Bladder Continence	37.75 (6.04) 8	41.40 (7.90) 30	37.18 (7.87) 65	0.05 ^‡^ 2, 100	39.88 (9.34) 8	40.00 (8.44) 25	35.74 (7.31) 68	0.04 2, 98

Note: df = degrees of freedom; ANOVA = Analysis of Variance; ^a^ Bathing did not have enough residents in each category to power a one-way ANOVA; ^†^ *p* < 0.05 for mean difference between residents who were independent in ADL and residents who were completely dependent; ^‡^ *p* < 0.05 for mean difference between residents with modified independence in ADL and residents with complete dependence in ADL; ^§^ *p* < 0.05 for mean difference between residents who were independent in ADL and residents with modified dependence in ADL.

**Table 3:** QOL-AD mean scores across three levels of dependency for each FIM item.

Individual FIM ADL Items	Baseline QOL-AD Score								6-Months QOL-AD Score							
	Residents independent in ADL Mean (SD) *n*		Residents with modified dependence in ADL Mean (SD) *n*		Residents with complete dependence in ADL Mean (SD) *n*		ANOVA *p*-value df		Residents independent in ADL Mean (SD) *n*		Residents with modified dependence in ADL Mean (SD) *n*		Residents with complete dependence in ADL Mean (SD) *n*		ANOVA *p*-value df	
Eating	39.84 (7.54) 45		38.09 (7.97) 53		29.80 (5.762) 5		0.02 ^†^ 2, 100		41.21 (7.59) 24		37.29 (7.58) 59		31.11 (5.84) 18		<0.001 ^†,‡^ 2, 98	
Grooming	41.87 (6.38) 15		40.33 (7.39) 57		33.35 (7.287) 31		<0.001 ^†,‡^ 2, 100		45.09 (8.29) 11		38.78 (6.10) 49		33.00 (7.57) 41		<0.001 ^†,‡,§^ 2, 98	
Bathing	41.00 (7.62) 4		41.88 (7.52) 17		37.62 (7.89) 82		0.11 2, 100		45.50 (12.02) 2		43.41 (6.46) 17		35.61 (7.44) 82		<0.001 ^‡^ 2, 98	
Dressing Upper Body	43.36 (6.99) 14		39.60 (7.24) 52		35.00 (7.90) 37		0.001 ^†,‡^ 2, 100		45.31 (6.28) 16		37.63 (6.05) 43		33.48 (7.84) 42		<0.001 ^†,‡,§^ 2, 98	
Dressing Lower Body	43.54(6.44) 13		39.20 (8.01) 35		36.78 (7.71) 55		0.02 ^†^ 2, 100		47.78 (7.10) 9		39.16 (5.51) 25		34.93 (7.50) 67		<0.001 ^†,‡,§^ 2, 98	
Toileting	43.59 (4.98) 17		39.17 (8.11) 36		36.20 (7.80) 50		0.003 ^†^ 2, 100		45.36 (7.45) 11		39.26 (6.23) 38		33.81 (7.42) 52		<0.001 ^†,‡,§^ 2, 98	
Bladder Management	42.05 (7.77) 21		42.22 (5.33) 9		36.96 (7.82) 73		0.01 ^†^ 2, 100		45.07 (7.67) 15		36.39 (5.83) 18		35.56 (7.50) 68		<0.001 ^†,§^ 2, 98	
Bowel Management	42.00 (7.28) 22		40.77 (6.37) 13		36.87 (8.00) 68		0.02 ^†^ 2, 100		42.40 (8.52) 20		37.14 (6.09) 21		35.35 (7.62) 60		0.002 ^†^ 2, 98	
Transfers (Bed to Wheelchair/Chair)	39.37 (7.74) 49		37.85 (7.33) 41		36.92 (10.36) 13		0.51 2, 100		38.80 (7.91) 49		36.43 (6.67) 30		34.32 (8.93) 22		0.08 2, 98	
Transfers (Toilet)	40.76 (7.04) 59		34.00 (7.70) 29		38.00 (8.324) 15		0.001 ^§^ 2, 100		39.47 (7.48) 45		36.48 (6.83) 33		33.43 (8.96) 23		0.01 ^†^ 2, 98	
Transfers (Tub/Shower)	38.60 (6.58) 5		38.53 (8.63) 36		38.40 (7.71) 62		1.00 2, 100		41.33 (12.50) 3		37.90 (6.84) 42		36.30 (8.47) 56		0.40 2, 98	
Locomotion (Walking/Wheelchair)	39.34 (7.80) 74		38.56 (7.89) 16		33.31 (7.22) 13		0.04 ^†^ 2, 100		38.54 (7.92) 54		35.77 (7.46) 26		35.14 (8.19) 21		0.15 2, 98	
Locomotion (Stairs) ^a^	0 Residents		0 Residents		111 Residents		N/A		1 Resident		1 Resident		99 Residents		N/A	

Note: df = degrees of freedom; ANOVA = Analysis of Variance; ^a^ Locomotion (Stairs) did not have enough residents in each category to power a one-way ANOVA; ^†^ *p* < 0.05 for mean difference between residents who are independent and residents who were completely dependent; ^‡^ *p* < 0.05 for mean difference between residents with modified independence and residents who were completely dependent; ^§^ *p* < 0.05 for mean difference between residents who were independent in ADL and residents with modified dependence in ADL.

At baseline, six of nine items on the RAI-MDS (not including Transfers, Bathing and Bladder Continence) had higher mean QOL-AD scores in the higher independence categories; however these differences were not statistically significant. At six months, five of these six items were statistically significant, with higher QOL-AD scores in the higher independence categories. Personal Hygiene and Toilet Use had the largest differences at six months (*p* ≤ 0.003). The mean QOL-AD difference for Personal Hygiene was 11.32 between independent residents (mean = 47.00, SD = 12.08) and those who were completely dependent (mean = 35.68, SD = 7.38; *p* = 0.001). Similarly, there was a 9.66 mean difference in QOL-AD between residents who were independent in Toilet Use (mean = 45.42, SD = 9.68) and those who were completely dependent (mean = 35.77, SD = 7.67; *p* = 0.003). Sample sizes ranged from 2 to 69 at baseline and 4 to 80 at six months. Results of these one-way ANOVAs, with the RAI-MDS items as the independent variables, are reported in Table 2.

At baseline and six months, nine of 13 FIM items had a statistically significant difference in QOL-AD means. Four of those nine items had significant differences between all three levels of dependency at six months. The largest differences were observed for Grooming and Dressing Upper Body: QOL-AD means between residents Independent and Completely Dependent in Grooming were 8.52 at baseline and 12.09 at six months. Similarly, the differences in levels of dependency for Dressing Upper Body were 8.36 at baseline and 11.83 at six months. Sample sizes ranged from 4 to 82 at baseline and 2 to 82 at six months. Results of these one-way ANOVAs, with FIM items as the independent variables, are reported in Table 3.

### 3.3. Health-Related Quality of Life Regression Models

Gender was not adjusted for in the regression because it did not meet the requirement of a coefficient change of less than <0.20. Age, cognition, depression and medical instability met the coefficient change criteria and were included in the final regression models (Table 4).

**Table 4:** Adjusted Regressions of ADL Scales with QOL-AD at Baseline and 6-Months.

ADL Scales	Baseline				6-Months			
	Beta	*p*-Value	Intercept	Confidence Intervals	Beta	Intercept	*p*-Value	Confidence Intervals
ADL Short	−0.064 *	0.535	45.3	(−0.6, 0.3)	−0.179 ^‡^	50.1	0.079	(−1.0, 0.1)
ADL Long	−0.071 *	0.478	45.5	(−0.4, 0.2)	−0.151 ^‡^	49.7	0.122	(−0.5,0.1)
ADL Hierarchy	−0.086 *	0.378	46.2	(−2.2, 0.8)	−0.144 ^§^	50.6	0.136	(−2.7, 0.4)
FIM Total	0.459 ^†^	<0.001	15.5	(0.1, 0.3)	0.487 ^†^	23.3	<0.001	(0.1, 0.3)

* CPS score and DRS score were included in the final regression models; ^†^ CPS score, DRS score, CHESS score, Age were included in the final regression models; ^‡^ CPS score, DRS score, and CHESS score were included in the final regression model; ^§^ CPS score and CHESS score were included in the final regression model.

A bivariate linear regression analysis of QOL-AD scores with RAI-MDS ADL Short scores indicated that greater independence in ADL Short at baseline (β = −0.064, confidence intervals: −0.6, 0.3) and six months (β = −0.179, confidence intervals: −1.0, 0.1) explained a higher HRQL score. The same trend was seen with ADL Long scores at baseline (β = −0.071, confidence intervals: −0.4, 0.2) and six months (β = −0.151, confidence intervals: −0.5, 0.1). The RAI-MDS ADL Hierarchy scale demonstrated an effect on HRQL at baseline and at six months. After adjusting for confounders, the RAI-MDS ADL scale regressions were not statistically significant at both time points. (Table 4).

The bivariate linear regression of QOL-AD and FIM total scores showed a large positive relationship at both baseline (β = 0.459, confidence intervals: 0.1, 0.3) and six months (β = 0.487, confidence intervals: 0.1, 0.3) (see Table 4).

### 3.4. Pearson’s Correlation

Dressing, Eating, and Toilet Use in the RAI-MDS and Eating, Grooming, Bathing Dressing Upper and Lower Body, Toileting, Bladder and Bowel Management, and Toilet Transfers in the FIM were the only items that were significantly correlated at both time points (Table 5). Within the RAI-MDS data, Transfer and Walk in Room were never significantly correlated with HRQL and neither was Transfers (Tub/ Shower) within the FIM.

**Table 5:** Correlation of QOL-AD scores and individual RAI-MDS and FIM item scores.

	Pearson Correlation Coefficient at Baseline		Pearson Correlation Coefficient at Six Months	
RAI-MDS 2.0 Items	*r*	*p*-value	*r*	*p*-value
Transfer	−0.08	0.45	−0.15	0.14
Walk in Room	0.02	0.83	0.12	0.25
Dressing	−0.24	0.02	−0.41	<0.001
Eating	−0.22	0.02	−0.29	0.003
Toilet Use	−0.22	0.02	−0.44	<0.001
Personal Hygiene	−0.17	0.1	−0.43	<0.001
Bathing	−0.17	0.09	−0.23	0.020
Bowel Continence	−0.13	0.19	−0.25	0.01
Bladder Continence	−0.17	0.09	−0.26	0.01
**FIM Items**	***r***	***p*-value**	***r***	***p*-value**
Eating	0.25	0.01	0.41	<0.001
Grooming	0.44	<0.001	0.44	<0.001
Bathing	0.24	0.01	0.42	<0.001
Dressing Upper Body	0.39	<0.001	0.51	<0.001
Dressing Lower Body	0.30	0.002	0.48	<0.001
Toileting	0.40	<0.001	0.46	<0.001
Bladder Management	0.28	0.005	0.38	<0.001
Bowel Management	0.29	0.003	0.34	<0.001
Transfers (Bed to Wheelchair/Chair)	0.16	0.10	0.26	0.01
Transfers (Toilet)	0.24	0.02	0.28	0.004
Transfers (Tub/Shower)	−0.01	1.0	0.19	0.06
Locomotion (Walking/Wheelchair)	0.26	0.01	0.12	0.22
Locomotion (Stairs)^a^	n/a	n/a	n/a	n/a

^a^ Locomotion (Stairs) had almost all residents score a 1 (total assistance) at both time points so a correlation was not performed.

## 4. Discussion

The results demonstrate that basic ADL such as dressing, eating and toilet use had a reasonably positive association with HRQL: Greater independence in ADL was associated with higher HRQL scores in nursing home residents with dementia. Walking and transfers did not have a significant association with HRQL in our sample. Perhaps this is because only a small proportion of participants (34%) were able to walk at baseline. The ability to groom and dress the upper body may be indicators of dignity, which is linked to HRQL [39]. Similarly, incontinence and depression have been associated with lower HRQL scores [40].

Our findings are consistent with the findings of others. A systematic review of factors associated with HRQL in residents with dementia found a negative relationship between ADL dependence and HRQL ratings in bivariate analyses but ambiguous results in multivariate analysis [41]. Similarly, after adjusting for confounders in a multivariate regression, only the FIM ADL scale was statistically significant at both time points while the RAI-MDS scales were not significant. Yeaman *et al.* [14] found a small positive correlation between QOL-AD scores in veterans with Alzheimer’s disease and independence in bathing, feeding, dressing, toileting, continence, and transferring. Others have found a significant correlation between HRQL and ADL in a sample of frail older adults [15] and suggest focusing on interventions that improve ADL to improve HRQL of older adults with limited physical functioning [16,42].

Generally, not as many factors of the RAI-MDS were significant compared with the number of FIM domains. This was seen as well with the ADL scales. Nonetheless, both the RAI-MDS and the FIM exhibited the same trend in associations between the individual items and total scores. A possible explanation for the difference may be that the FIM and QOL-AD were gathered at the same time in our study, by the same people (research assistants and health care aides providing direct care to residents), in a similar manner (proxy interviews); whereas the RAI-MDS assessments were completed once every quarter, in accordance with routine facility schedules, by nursing home staff who also rely on proxy reports from direct care providers. Reliability of the data may be questioned because staff members completing the assessment complete it for funding and administrative purposes rather than for research purposes. Furthermore, changes in the resident may go undetected between routine assessments. The RAI-MDS may reflect more HRQL domains and could have other confounders. Attempting to use the RAI-MDS as a surrogate measure appears suboptimal as such issues undermine the reliability of the data. The RAI-MDS should probably not be used in this manner to shape institutional care plans as this may impair quality of care.

As with all studies, this study has limitations. The original quasi-experimental study did not involve randomization. While we have no reason to believe that this sample of participants was unusual, generalizing beyond this study must be done cautiously. Although many factors influence HRQL, we focused solely on the ADL component; therefore future research is recommended to strengthen the association of ADL and HRQL by accounting for other covariates.

Another limitation of this study is that the FIM and HRQL data were gathered from proxy sources, which may have led to over or underestimating HRQL scores [43]. Literature suggests that proxy rating accuracy can be influenced by various factors such as resident and proxy socio-demographic characteristics, frequency of contact, and experience of health care provider, but the findings are inconsistent [43]. The resident may deem non-ADL factors as being more important to their quality of life even as their ADL function declines over time due to nursing home admission, the types of anti-dementia and anti-psychotic drugs used, and/or the decline in mobility with age and comorbidities [44,45,46]. Using a person-centred approach by asking the person with dementia to report their HRQL would move us closer to being able to assess the nursing home residents’ perceptions of their own quality of life. Unfortunately, this is a complex issue to address because there is usually a decline in communication function as the dementia progresses associated with more severe aphasia and leading to the inability to access the perspective of the person with dementia [47]. This was seen with the development of the QOL-AD measure. The original version allowed for dialogue between the community-dwelling individual with dementia, however the measure was later adapted to a proxy questionnaire for nursing home residents who usually have more advanced dementia [18]. Although it is possible that some residents in our study may have been able to use a self-administered HRQL measure; proxy assessments were used to standardize all of the assessments. Furthermore, if a self-administered HRQL measure was used at baseline, the progression of dementia with some participants could have led to missing data at the second data collection point [7].

We found that ADL function is an integral component of HRQL for people with dementia. Given that in 2010 there were 35.6 million people living with dementia worldwide, and that this number is expected to double every 20 years [48], increasing dependency in people with dementia poses a significant social, psychological and financial burden on caregivers and health care systems. The widespread international use of the FIM makes it a potential data source for indicators of HRQL in future research [11,49]. Using resources already in place, such as the FIM, it is an opportunity to leverage existing data to inform future quality improvement initiatives to guide important HRQL outcomes for people with dementia. Optimizing independence in basic ADL will have important implications for reducing nursing care requirements in activities such as bathing, dressing, toileting, transferring and eating. Future longitudinal research, particularly with the FIM, could continue to examine the relationship between ADL dependency and HRQL to understand the benefit of promising practice initiatives that focus on ADL function and the subsequent effect on HRQL.

## 5. Conclusions

The present study demonstrates there is an association between independence in activities of daily living, such as toileting and personal hygiene, and higher health-related quality of life in nursing home residents with dementia. This relationship is strongly supported by the FIM. Thus, with future longitudinal research, the FIM has potential to be a surrogate measure for quality of life while the widespread use of the FIM in facilities increases the utility of findings.
